# Cognitive Improvement in Children With Cerebral Palsy Following Combined Scalp Acupuncture and High-Frequency Repetitive Transcranial Magnetic Stimulation (rTMS): A Prospective Observational Study

**DOI:** 10.7759/cureus.87672

**Published:** 2025-07-10

**Authors:** Xiao Yu Shen, Zhen Zhen Zhao, Wei Yi Huang, Jian Guo Zhong

**Affiliations:** 1 Rehabilitation, The Second Affiliated Hospital of Chengdu Medical College (China National Nuclear Corporation 416 Hospital), Chengdu, CHN

**Keywords:** cerebral palsy, cognitive improvement, high-frequency rtms, rehabilitation, scalp acupuncture

## Abstract

Objective: To evaluate the clinical efficacy of combining scalp acupuncture and high-frequency repetitive transcranial magnetic stimulation (rTMS) for improving cognitive function in children with cerebral palsy (CP) and cognitive impairment.

Methods: In this prospective observational study, 60 children with CP and cognitive impairment were recruited from January 2023 to June 2024 at a tertiary rehabilitation center. Participants were divided into two groups (n=30 each): the observation group received scalp acupuncture plus high-frequency rTMS (3 sessions/week), while the control group received scalp acupuncture alone (same frequency). Both groups underwent concurrent routine rehabilitation (150 minutes/session, three sessions/week) for 24 weeks. Cognitive function was assessed using WISC-IV before and after intervention. Statistical analyses included t-tests, chi-square tests, and non-parametric tests.

Results: Baseline characteristics (age, gender, and disease duration) showed no differences between the two groups (p>0.05). Pre-intervention, compared with the control group, total WISC scores were higher (64.74±4.00 vs. 60.07±4.54, p＜0.001), and a greater proportion of children with mild cognitive impairment (25/30 vs. 14/30, p=0.037) were in the observation group. Post-intervention, compared with the control group, total WISC scores were higher (75.03±4.72 vs. 65.90±4.81, p＜0.001), 65.90±4.81，p＜0.001), mean change scores were increased (10.57±2.81 vs. 5.83±1.70, p＜0.001), the Perceptual Reasoning Index was higher (82.26±7.62 vs. 74.16±10.31, p＜0.001), and the Working Memory Index was higher (78.90±7.12 vs. 67.33±7.70, p＜0.001) in the observation group.

Conclusion: The combination of scalp acupuncture and high-frequency rTMS significantly enhances cognitive function in children with CP and cognitive impairment compared to scalp acupuncture alone.

## Introduction

Cerebral palsy (CP) represents a spectrum of neurodevelopmental disorders primarily characterized by movement impairments, with a global incidence ranging from 1.6 to 2.37 per 1000 live births [1-3]. Notably, cognitive impairment affects 30-58% of children with CP, manifesting as intellectual disability, language delays, learning difficulties, executive dysfunction, and social adaptation challenges [4,5]. These cognitive deficits significantly compromise patients' activity participation and impose substantial socioeconomic burdens, making cognitive rehabilitation a critical priority in long-term CP management.

Scalp acupuncture has emerged as a cornerstone intervention in CP rehabilitation due to its neuromodulatory effects [6]. This modality operates through the meridian system connecting scalp acupoints with visceral functions, demonstrating particular efficacy for cognitive dysfunction. Our clinical practice primarily employs Micro-system Scalp-points (MS6 and MS7), which show excellent patient compliance. While this technique offers practical advantages in administration, its cognitive benefits remain limited, necessitating complementary approaches.

Recent advances in physiotherapy have established repetitive transcranial magnetic stimulation (rTMS) as a promising non-invasive neuromodulation technique for CP [7]. By delivering targeted cortical stimulation, rTMS can directly modulate central nervous system plasticity, offering distinct advantages in neurorehabilitation. The high-frequency rTMS protocol has shown particular potential for enhancing cognitive outcomes in pediatric populations.

To investigate synergistic therapeutic effects, this prospective observational study compares two intervention protocols: 1) conventional scalp acupuncture alone versus 2) combined scalp acupuncture and high-frequency rTMS. Using the Wechsler Intelligence Scale as a general measure of cognitive ability, we evaluated cognitive changes following 24 weeks of treatment. Our findings aim to establish an evidence base for standardizing rTMS applications in CP cognitive rehabilitation while informing the design of future randomized controlled trials.

## Materials and methods

Participants

This prospective study enrolled 60 pediatric patients with cerebral palsy combined with cognitive impairment from the Department of Rehabilitation Medicine at the Second Affiliated Hospital of Chengdu Medical College (China National Nuclear Corporation 416 Hospital) from January 2023 to June 2024. Participants were assigned to groups based on clinical referral order, with sequential enrollment ensuring naturalistic observation. The participants were equally allocated into two groups (n=30 each) through a prospective observational design. The schedule of enrollment, interventions, and assessments was included in Table 1.

**Table 1:** Schedule of enrollment, interventions, and assessments rTMS: repetitive transcranial magnetic stimulation, WISC: the Wechsler intelligence scale for children

	Study period					
	Enrollment	allocation	Control group		Observation group	
			Pre-intervention	Post-intervention	Pre-intervention	Post-intervention
							Enrollment						
Eligible screen	√					
Informed consent	√					
allocation		√				
Interventions						
							Scalp acupuncture			√	√	√	√
High-frequency rTMS					√	√
Assessments						
WISC			√	√	√	√
							Adverse events				√		√

Diagnostic criteria

Confirmed CP diagnosis per the Chinese Cerebral Palsy Rehabilitation Guidelines (2022) [8].

Inclusion criteria

1) WISC-IV scores between 50 and 69 points; 2) Age 7-18 years; 3) Legal guardian-provided informed consent.

Exclusion criteria 

1) Non-CP-related cognitive impairment; 2) Combined epilepsy; 3) Severe organic disorders; 4) Inherited metabolic diseases.

Ethical considerations 

The study was approved by the Ethics Committee of Nuclear Industry 416 Hospital (KJ2022021) and conducted in accordance with the Declaration of Helsinki principles.

Study design

This study employed a non-randomized, real-world prospective observational design, in which participants were allocated into two groups based on the actual rehabilitation treatment received: 1) scalp acupuncture alone therapy versus 2) combined scalp acupuncture and rTMS therapy. The grouping strategy was based on actual clinical rehabilitation protocols received by patients, offering several methodological advantages and limitations.

Sample size justification

The sample size (n = 30 per group) was determined based on clinical feasibility and a review of related intervention studies. Although no formal power analysis was conducted, previous studies using WISC-IV scores suggest that an effect size of approximately 0.8 (Cohen’s d) with α= 0.05 and 80% power would require approximately 26 participants per group, supporting the adequacy of our sample size. This is consistent with findings from studies evaluating cognitive interventions in children with neurological conditions, which often report medium to large effect sizes when using the WISC-IV as an outcome measure [9-11].

Grouping and treatment

Both groups underwent a standardized rehabilitation training comprising motor function training (120 minutes/session, 3 sessions/week) including progressive resistance stretching, active assistive joint mobility exercises, dynamic core stabilization, and graded balance coordination drills, alongside cognitive therapy (30 minutes/session, 3 sessions/week) consisting of game-based occupational therapy interventions targeting visual-spatial processing through shape/color identification tasks, working memory via alphanumeric sequencing exercises, pattern completion/matching activities, and temporal-spatial orientation training, with all interventions administered by certified therapists to ensure treatment fidelity while maintaining consistent intensity across groups through evidence-based, age-appropriate approaches that simultaneously addressed both motor and cognitive domains.

Control group 

On the basis of routine rehabilitation training, the patient received scalp acupuncture treatment; the acupuncture site was selected from the parietal anterior temporal oblique lines (Micro-system Scalp-points, MS6) and parietal posterior temporal oblique lines (Micro-system Scalp-points, MS7) on the affected side. Disposable sterile acupuncture needles (0.25*25 mm) were used to puncture under the scalp in segments along the top of the head towards the side of the head, and the needles were left in place for 30 minutes after obtaining qi (30 minutes/session, three sessions/week).

Observation group

The scalp acupuncture treatment method is as above. The transcranial magnetic stimulator (Shenzhen Yingzhi Technology Co., Ltd.) was used to measure the motion threshold (MT) before the initial treatment, and the child was placed in a seated position, and the M1 motor cortex was stimulated with a figure-of-eight coil for 10 consecutive stimulations, with the intensity of stimulation being gradually reduced, and the motion-evoked potentials (MEP) generated by the contraction of the contralateral bunion were collected, and the MEP was measured. During treatment, the child wore a positioning cap on the head and was positioned in the sitting or recumbent position, and the figure-of-eight coil was placed on the right dorsolateral prefrontal cortex of the child, with a stimulation intensity of 90%-100% rMT and a stimulation frequency of 5 Hz at 8-s intervals for 20 s. The stimulation frequency was 5 Hz. 20s each time, 8s interval, total number of pulses 1000 (3 sessions/week). The rTMS target region was selected due to its established role in cognitive regulation in pediatric CP, according to the Chinese Cerebral Palsy Rehabilitation Guidelines (2022). All rTMS sessions were administered by the same clinician.

Adverse events

No adverse events were reported during the course of rTMS or scalp acupuncture treatment. Safety was monitored through parent reports and therapist logs throughout the study period.

Measurements

**Figure 1:**
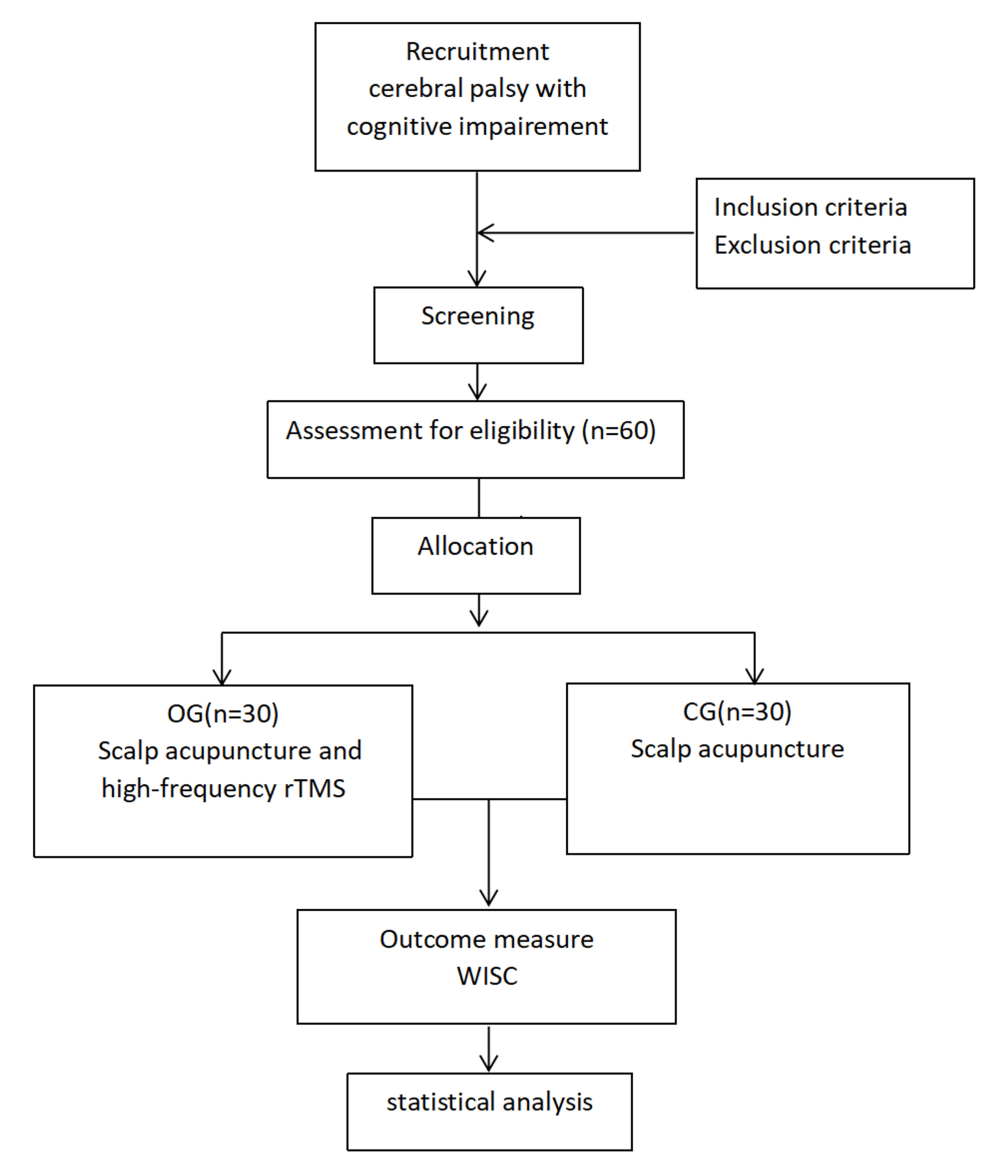
Study flow chart OG: observation group, CG: control group, WISC: the Wechsler intelligence scale for children

Cognitive functions were assessed using the Wechsler Intelligence Scale for Children-The Fourth Vision (WISC-IV) before and after 24 weeks of treatment, respectively. The Wechsler Intelligence Scale for Children (WISC) consists of 14 items, which are categorized into 4 indexes: Verbal Comprehension Index (VCI), Perceptual Reasoning Index (PRI), Working Memory Index (WMI), and Processing Speed Index (PSI). VCI includes similarities, vocabulary, comprehension, and information. PRI includes block design, picture concepts, matrix reasoning, and picture completion. WMI includes digit span, letter-number sequencing, and arithmetic. PSI includes coding, symbol search, and cancellation. Completion of at least 10 items is required. The evaluator calculated the raw scores, then entered them into the King May Psychological Assessment [29]. The system automatically generated the composite scores, which were finally used for data analysis. King May Psychological Assessment Technology Development Ltd. is authorized by Pearson Clinical Assessments, APAC & India. The evaluator was qualified to use the Wechsler Intelligence Scale for Children, Fourth Edition, Chinese version by King May Psychological Assessment, and the license number is No. 010903613. The flowchart is in Figure 1.

Data statistics

IBM Corp. Released 2017. IBM SPSS Statistics for Windows, Version 26.0. Armonk, NY: IBM Corp. was used for data analysis. Measurement data were described by mean ± SD, and count data were described by percentages. According to the normality or otherwise of the data, a paired-sample t-test or non-parametric test was used for within-group comparisons, and an independent-sample t-test, chi-square test, non-parametric test, Mann-Whitney U test, ANOVA, etc., were used for between-group comparisons. Differences were considered statistically significant at p < 0.05.

## Results

There were no significant differences between the observation and control groups in terms of age, sex distribution, or disease duration (p > 0.05), indicating comparability between the two groups. Compared with the control group, at baseline, the observation group demonstrated significantly higher scores on the total WISC scale (p < 0.001), Perceptual Reasoning Index (PRI) (p = 0.024), and Working Memory Index (WMI) (p = 0.001). There were no significant group differences in Verbal Comprehension Index (VCI) or Processing Speed Index (PSI) (p > 0.05). The basic information of the patients in the 2 groups is shown in Table 2.

**Table 2:** Basic information of the two groups *Compared with control group, p＜0.05; WISC: Wechsler Intelligence Scale, VCI: Verbal Comprehension Index, PRI: Perceptual Reasoning Index, WMI: Working Memory Index, PSI: Processing Speed Index

Items	Observation group	Control group	Statistical value（t/F/U/c2）	P
	（n=30）	（n=30）		
Age year）	8.87±1.17	9.20±2.12	-0.19	0.85
Sex (%)			0.067	0.795
Male	16（53.33%）	13（43.33%）		
Female	14（46.67%）	17（56.67%）		
Duration (year)	6.27±2.07	6.37±2.66	-0.33	0.741
WISC	64.73±4.00*	60.07±4.54	-3.739	0
VCI	68.50±7.30	65.67±8.11	1.422	0.16
PRI	75.23±9.66*	69.03±11.08	2.31	0.024
WMI	68.40±9.78*	60.30±10.85	-3.22	0.001
PSI	65.20±7.67	63.53±8.29	-0.934	0.35

Compared with pre-treatment, WISC scores increased in both groups (p < 0.01). Compared with the control group, the WISC scores of the observation group before and after treatment were higher (p < 0.01), and the mean change scores of the observation group after treatment were higher by about 1.8 times (p < 0.05) (Table 3).

**Table 3:** Comparison of WISC scores between the two groups pre-treatment and post-treatment #Compared with pre-treatment, p＜0.05, *Compared with control group, p＜0.05 WISC: Wechsler Intelligence Scale

Groups	Numbers	WISC		
		Pre-treatment	Post-treatment	Mean change scores
Observation group	30	64.73±4.00*	75.30±4.72#*	10.57±2.81*
Control group	30	60.07±4.54	65.90±4.81#	5.83±1.70
U value		-3.739	-5.403	-5.788
p-value		0.000	0.000	0.000

Pre-treatment, compared with the control group, mild cognitive impairment patients were more in the observation group (c2=8.864, p=0.003). Post-treatment, compared with the control group, the number of patients in the critical state of cognitive dysfunction was higher in the observation group (c² = 13.726, p = 0.001) (Table 4).

**Table 4:** Comparison of severity of cognitive dysfunction between the two groups pre-treatment and post-treatment *Compared with the control group, p＜0.01 WISC: Wechsler Intelligence Scale

Treatment period	Groups	WISC				
		50-59	60-69	70-79	80-89	total
Pre-treatment	Observation group	5	25*	0	0	30
	Control group	16	14	0	0	30
c2 value	8.864					
P-value	0.003					
Post-treatment	Observation group	0	7	17*	6	30
	Control group	2	21	7	0	30
c2 value	13.726					
P-value	0.001					
Notes: *Compared with control group，p＜0.01						

Compared with the control group, the Perceptual Reasoning Index and Working Memory Index increased more significantly after treatment in the observation group (p < 0.01); there was no statistically significant difference in the changes of the Verbal Comprehension Index and Processing Speed Index (p > 0.05) (Table 5).

**Table 5:** Comparison of Indexes of WISC between the two groups post-treatment *Compared with the control group, p＜0.01 WISC: Wechsler Intelligence Scale, VCI: Verbal Comprehension Index, PRI: Perceptual Reasoning Index, WMI: Working Memory Index, PSI: Processing Speed Index

Groups	Numbers	WISC			
		VCI	PRI	WMI	PSI
Observation group	30	76.57±8.27	82.26±7.62*	78.90±7.12*	71.80±6.60
Control group	30	69.87±7.63	74.16±10.31	67.33±7.70	67.03±7.93
t/U value		3.262	-3.516	-5.344	2.530
p-value		0.679	0.000	0.000	0.137

## Discussion

Cognitive dysfunction is a significant comorbidity in children with cerebral palsy (CP), resulting from non-progressive developmental brain injuries that lead to irreversible neuroanatomical changes. Notably, damage to the striato-parietal junction has been identified as a critical neuropathological basis for cognitive impairment in this population [12,13]. Cognitive function encompasses the brain’s integrated capacity for information processing, comprehension, and problem-solving through complex neurophysiological mechanisms. When impaired, it manifests as a heterogeneous array of clinical symptoms [14].

In pediatric CP, cognitive dysfunction commonly affects four principal domains: (1) attention, (2) visuospatial perception, (3) memory, and (4) executive function [15]. Current diagnostic protocols rely on a combination of neuropsychological assessments and electrophysiological measures [16], with the Wechsler Intelligence Scale for Children (WISC) widely regarded as the gold standard. The WISC is distinguished by its multidimensional framework, comprising 14 subtests and four composite indices that generate a total IQ score, thus enabling a comprehensive evaluation of cognitive profiles [17].

Advanced neuroimaging and neurophysiological modalities, such as event-related potentials (e.g., P300), structural and functional magnetic resonance imaging (MRI), diffusion tensor imaging (DTI), and functional near-infrared spectroscopy (fNIRS), have shown high sensitivity in detecting cognitive impairment [18-21]. Nevertheless, their broader clinical utility is constrained by issues related to technical complexity, lack of standardization, and high cost. Therefore, the present study adopted the WISC as the primary outcome measure due to its validated reliability, feasibility in clinical practice, and widespread use in evaluating therapeutic outcomes in CP-related cognitive impairment.

Scalp acupuncture has been demonstrated to improve language deficits, intellectual disability, and gross motor function in children with CP. It is classified as a Grade B recommended intervention in the Chinese Cerebral Palsy Rehabilitation Guidelines (2022) [22]. According to traditional Chinese medicine (TCM) theory, scalp acupuncture facilitates cerebral function by dredging meridians, regulating Yin-Yang and Qi-blood balance, and nourishing the brain and sensory orifices. Acupoints are selected based on TCM pattern identification and cortical functional localization theories, commonly including Baihui, Sishencong, Shenting, Naohu, and the Three Wisdom Needles. Specific cortical zones such as the motor, balance, sensory, speech, and visual areas can be targeted to enhance therapeutic outcomes [23,24]. Although scalp acupuncture is technically straightforward, its efficacy is highly dependent on correct theoretical guidance and flexible point selection. In this study, we employed the parietal anterior temporal oblique line and parietal posterior temporal oblique line on the affected side, an approach routinely utilized in our department with proven clinical efficacy in previous studies.

Transcranial Magnetic Stimulation (TMS) has emerged as a promising non-invasive brain stimulation technique in the rehabilitation of children with CP, owing to its unique ability to directly modulate cortical activity [25,26]. Unlike conventional rehabilitation or scalp acupuncture, TMS delivers magnetic pulses that penetrate the skull without attenuation, regulating cortical excitability and corticospinal tract function, enhancing neuroplasticity, and promoting synaptic reorganization [27,28]. However, as TMS becomes more widespread in China, concerns have arisen over non-standardized procedures, inconsistent parameter settings, inaccurate targeting, and suboptimal therapeutic environments. The 2022 Expert Consensus on Transcranial Magnetic Stimulation Therapy for Children with Cerebral Palsy provides structured guidelines for clinical application, offering a reliable reference for standardizing treatment protocols.

This study employed a prospective observational design. Participants were grouped based on their actual rehabilitation interventions: the control group received conventional rehabilitation combined with scalp acupuncture, while the observation group received both scalp acupuncture and TMS. The scalp acupuncture technique used was consistent with established protocols in our department. The TMS protocol followed the recommendations from the 2022 Expert Consensus, targeting the right dorsolateral prefrontal cortex using repetitive TMS (rTMS) to enhance cognitive function.

Study findings revealed that, at baseline, the WISC scores, specifically the total IQ, Perceptual Reasoning Index, and Working Memory Index, were significantly higher in the observation group compared to the control group. Subgroup analyses further showed that cognitive deficits in the observation group were generally milder. Following treatment, these cognitive indices improved more markedly in the observation group. After 24 weeks, both groups demonstrated increased total IQ scores, the mean improvement in the observation group was approximately 1.8 times that of the control group, the 10.57-point improvement in observation group was associated with a shift in cognitive classification from "moderate impairment" to "mild impairment" or even "borderline normal" in several participants, suggesting functional relevance in academic and adaptive domains.

Despite the encouraging results, several limitations of the study design must be acknowledged. First, the lack of randomization may introduce selection bias, as evidenced by higher baseline cognitive scores in the observation group. Although this reflects a real-world clinical assignment, it limits causal inference. Future studies should adopt randomized controlled designs to reduce potential bias. Second, the study lacked blinding of participants, which may result in expectancy bias. In the study, we applied blinding to the assessors to reduce assessment bias. In pediatric rehabilitation, blinding is often challenging, but efforts should be made to minimize subjective influence. Third, although all participants received routine rehabilitation training, individual variation in therapy adherence and intensity may still exist. We attempted to mitigate this by standardizing protocols and monitoring therapist fidelity.

## Conclusions

The study demonstrated that scalp acupuncture combined with high-frequency repetitive transcranial magnetic stimulation (rTMS) can effectively improve cognitive function in children with cerebral palsy and comorbid cognitive impairment. The observed improvement was significantly greater than that achieved with acupuncture alone, indicating promising therapeutic potential and warranting further investigation. Importantly, the magnitude of cognitive improvement, particularly in perceptual reasoning and working memory, translates into enhanced functional abilities that may support better academic performance and daily adaptive functioning. This adds real-world value beyond statistical significance. The future research should explore the long-term sustainability of these effects through follow-up assessments and consider cost-effectiveness and accessibility in broader clinical implementation.

## Figures and Tables

**Figure 2:**
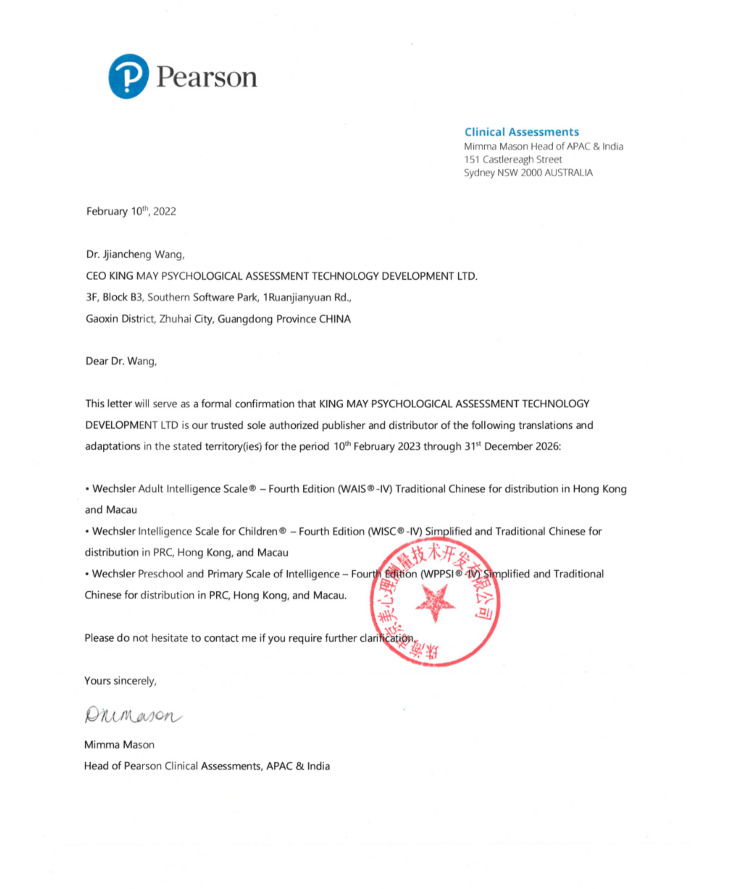
Certification for WISC
